# THE IMPORTANCE OF MAINTAINING PHYSICAL ACTIVITY FOR TRANSITIONS BETWEEN COGNITIVE STATES: A COORDINATED ANALYSIS

**DOI:** 10.1093/geroni/igz038.3323

**Published:** 2019-11-08

**Authors:** Tomiko Yoneda, Jonathan Rush, Nathan A Lewis, Jamie E Knight, Jinshil Hyun, Andrea Piccinin, Graciela Muniz Terrera

**Affiliations:** 1 University of Victoria, Victoria, British Columbia, Canada; 2 Albert Einstein College of Medicine, New York, New York, United States; 3 University of Victoria, Victoria, B.C., United States; 4 University of Edinburgh, Edinburgh, United Kingdom

## Abstract

Although existing research shows that physical activity (PA) protects against cognitive decline, it is unclear if maintenance of PA throughout older adulthood influences the timing of onset or transitions through cognitive states. Further understanding of modifiable lifestyle factors that protect against cognitive changes characteristic of both normal aging and pathological aging, such as Alzheimer’s disease and other dementias, is imperative. Data were drawn from fourteen longitudinal studies of aging from Europe and America (total N=53,069). Controlling for demographics and chronic conditions, multi-state models were independently fit between datasets to investigate the impact of PA (computed based on Metabolic Equivalent of Task Method) on the likelihood of transitioning through three cognitive states, while also accounting for death as a competing risk factor. Random effects meta-analysis of transition probabilities indicated that more PA was associated with a reduced risk of transitioning from normal cognition to mildly impaired cognition (HR=0.90, CI’s=0.84, 0.97, p=0.007) and death (HR=0.24, CI’s=0.06, 0.92, p=0.04), as well as an increased likelihood of transitioning from severe impairment back to mild impairment (HR=1.09, CI’s=1.01, 1.17, p=0.03). Engagement in national minimum recommendations for PA (~150 minutes/week) increased total life expectancy for 70 year old males and females by 4.08 and 5.47 years, respectively. These results suggest that engaging in at least 150 minutes of physical activity per week in older adulthood contributes to delays in onset of mild cognitive impairment, substantially increases life expectancy, and may also diminish the symptoms that contribute to poor cognitive performance at the severely impaired stage.

